# Persistent Hyperglycemia Is Associated With Increased Mortality After Intracerebral Hemorrhage

**DOI:** 10.1161/JAHA.117.005760

**Published:** 2017-08-02

**Authors:** Teddy Y. Wu, Jukka Putaala, Gagan Sharma, Daniel Strbian, Turgut Tatlisumak, Stephen M. Davis, Atte Meretoja

**Affiliations:** ^1^ Department of Medicine and Neurology Melbourne Brain Centre at the Royal Melbourne Hospital University of Melbourne Australia; ^2^ Department of Neurology Helsinki University Hospital Helsinki Finland; ^3^ Department of Radiology at the Royal Melbourne Hospital University of Melbourne Australia; ^4^ Department of Clinical Neurosciences Institute of Neuroscience and Physiology Sahlgrenska Academy at the University of Gothenburg Gothenburg Sweden; ^5^ Department of Neurology Sahlgrenska University Hospital Gothenburg Sweden

**Keywords:** edema, glucose, hyperglycemia, intracerebral hemorrhage, mortality, Intracranial Hemorrhage

## Abstract

**Background:**

Hyperglycemia may be associated with worse outcome after intracerebral hemorrhage (ICH). We assessed the association of early glycemic trajectory on ICH mortality and edema growth.

**Methods and Results:**

We included patients from the Helsinki ICH study with glucose measurements at least once between both 0 to 24 and 24 to 72 hours from onset. Hyperglycemia was defined as blood glucose ≥8 mmol/L (144 mg/dL) based on the local threshold for treatment. Glycemic trajectory was defined on maximum values 0 to 24 and 24 to 72 hours after ICH: (1) persistent normoglycemia in both epochs; (2) late hyperglycemia (only between 24 and 72 hours); (3) early hyperglycemia (only before 24 hours); and (4) persistent hyperglycemia in both epochs. Logistic regression with known predictors of outcome estimated the association of glycemic trajectory and 6‐month mortality. A generalized linear model assessed the association of glycemic trajectory and interpolated 72‐hour edema extension distance. A total of 576 patients met eligibility criteria, of whom 214 (37.2%) had persistent normoglycemia, 44 (7.6%) late hyperglycemia, 151 (26.2%) early hyperglycemia, and 167 (29.0%) persistent hyperglycemia. Six‐month mortality was higher in the persistent (51.1%) and early (26.3%) hyperglycemia groups than the normoglycemia (19.0%) and late hyperglycemia (3.6%) groups. Persistent hyperglycemia was associated with 6‐month mortality (odds ratio 3.675, 95% CI 1.989–6.792; *P*<0.001). Both univariate (*P*=0.426) and multivariable (*P*=0.493) generalized linear model analyses showed no association between glycemic trajectory and 72‐hour edema extension distance.

**Conclusion:**

Early hyperglycemia after ICH is harmful if it is persistent. Strategies to achieve glycemic control after ICH may influence patient outcome and need to be assessed in clinical trials.

**Clinical Perspective**

**What Is New?**

- Only half of intracerebral hemorrhage patients with baseline hyperglycemia remain persistently hyperglycemic.
- Association with mortality was only observed in patients with persistent hyperglycemia.

**What Are the Clinical Implications?**

- Optimizing glycemic status early after intracerebral hemorrhage may improve outcome after intracerebral hemorrhage.

## Introduction

The current American Stroke Association guidelines endorse avoidance of hyperglycemia in patients with intracerebral hemorrhage (ICH).^1^ The recommendation was based on association of hyperglycemia and poor outcome in observational studies.^2^, ^3^, ^4^, ^5^ The major limitation of these studies is use of single glucose measurement not accounting for potential glucose fluctuations after ICH. Six studies (n=60–295)^6^, ^7^, ^8^, ^9^, ^10^, ^11^ have utilized multiple glucose measurements in analysis of ICH outcome with mixed results. In preclinical studies, hyperglycemia increases neuronal cell death and brain edema by enhancing breakdown of the blood–brain barrier.^12^, ^13^ It is plausible that hyperglycemia could mediate secondary injury through similar mechanisms in human ICH.

There is emerging evidence that secondary injury from perihematomal edema is associated with poor ICH outcome.^14^, ^15^ Edema is strongly correlated with hematoma volume, but other mediators of edema growth including glucose are uncertain.^14^ It is possible that glucose and edema evolution are mechanistically related, making them potential modifiable therapeutic targets.

The aims of this study are to evaluate the impact of early glycemic trajectory on ICH mortality and edema growth. We hypothesized that persistent hyperglycemia is associated with increased mortality and edema growth.

## Methods

### Patients

Patients from the Helsinki ICH study^16^ (HICHS) were included. Briefly, HICHS is a retrospective analysis of 1013 consecutive ICH patients admitted to Helsinki University Hospital between January 2005 and March 2010. Data collection was performed retrospectively by chart review.^16^ Patients were excluded from the current analysis if there was no imaging data available, had missing 6‐month mortality data, or had no glucose measurement either within 24 hours and/or between 24 and 72 hours of ICH onset. Stroke onset time was determined from witnessed onset in 363 (63.0%) patients and from last known well time in 213 (37.0%) patients. Institutional approval for the study was granted by the Helsinki University Hospital, and patient consent was not required as there was no patient contact in this observational study registry.

### Planimetric ICH and Edema Volume Ascertainment

Hematoma and edema volumes were segmented using semiautomated planimetry.^17^ Briefly, de‐identified computed tomography images were loaded on Analyze 12.0 (Biomedical Imaging Resource; Mayo Clinic). Edematous regions were segmented using a fixed lower Hounsfield Unit (HU) of 5 and a flexible upper limit with a ceiling of 33 Hounsfield Units, comparing with the unaffected hemisphere for visual estimate of edema versus leukoaraiosis. For ICH, Hounsfield Unit range was kept within 44 to 100 Hounsfield Units. T.Y.W. performed segmentation on all scans. The segmented regions of interest were subjected to in‐house processing to derive volumes that take into account gantry tilt and true slice thickness as reported in detail elsewhere.^17^

### Glycemic Trajectory Determination

Hyperglycemia was defined as blood glucose ≥8 mmol/L (144 mg/dL) based on the local threshold for treatment. The Helsinki protocol during the study was to provide sliding scale insulin when the glucose was ≥8 mmol/L. Glycemic trajectory was defined on maximum values 0 to 24 and 24 to 72 hours after ICH. Four categories were determined: (1) persistent normoglycemia in both epochs; (2) late hyperglycemia (only between 24 and 72 hours); (3) early hyperglycemia (only before 24 hours); and (4) persistent hyperglycemia in both epochs.

### Edema Metric and Interpolated 72‐Hour Edema Volume

Edema extension distance (EED), a recently reported edema metric, was used in assessing the association of glycemic trends and edema growth.^18^ We have previously reported average edema growth trajectory derived from a negative exponential formula (EED growth=0.162×days^−0.927^, R^2^=0.82) utilizing data from HICHS.^19^ This growth rate equates to the average expected EED at any time point from ictus=2.210×days^0.07331^−1.478. As the patients were scanned at different time points in routine clinical practice, we interpolated the EED from the observed time of an individual's EED closest to 72‐hour time point, assuming the same proportional growth to derive the expected 72‐hour EED for the individual patient. In other words, if the patient's last scan was at 48 hours and was 70% of the average expected EED at this time point, we assumed the patients to also have 70% of average expected 72‐hour EED.

### Statistical Analysis

Standard descriptive statistics were used. Differences in patient characteristics, stroke severity, and imaging metrics between the glycemic trajectories were assessed on univariate analysis using χ^2^ or Fisher exact test for categorical variables and Mann–Whitney test for continuous variables. We used mortality at 6 months, the common time point of end point in ICH trials.^20^, ^21^ Survival differences between the glycemic trajectories were performed using the log rank test and plotted on Kaplan–Meier curve. Association with glycemic trend categories with 6‐month mortality was assessed in a logistic regression model adjusted for log‐transformed hematoma volume, age, male sex, prior warfarin use, baseline National Institutes of Health Stroke Scale (NIHSS), Glasgow Coma Scale (GCS), presence of intraventricular extension, male sex, and infratentorial location, which are factors associated with ICH outcome.^16^ Baseline ICH volume was log transformed before logistic regression analysis to avoid potential confounding by outliers. Collinearity between the covariates in the logistic regression model was checked. The model fit was tested using receiver operating characteristic area under the curve. The impact of glycemic trajectory on model fit was assessed by using a base model with log‐transformed ICH volume and age as covariates, a second model with all covariates other than glycemic trajectory, and finally the full model. In the secondary analyses, we repeated the primary analysis by replacing glucose trajectory with the maximum recorded glucose as continuous variable from each epoch (0–24 and 24–72 hours) adjusting for all predefined variables. Correlation between the maximum recorded glucose from the 2 epochs was assessed using Pearson correlation. Further, we assessed the association of absolute change in maximum glucose from 0 to 24 hours to 24 to 72 hours and mortality adjusted for 0 to 24‐hour glucose and other predefined covariates as listed above. In addition, the logistic regression analysis was also performed adjusting for hematoma growth (defined as 6‐mL growth or 33% relative growth on follow‐up computed tomography) in the subset of patients with follow‐up imaging available. Finally, we performed a propensity‐score matching analysis by estimating the propensity score for the covariates included in the primary logistic regression model. Matching was performed to the nearest neighbor with caliper set at 0.1 SD of the logit of the propensity scores. The primary analysis was then repeated in the propensity‐score‐matched population. Association between glycemic trajectory categories and interpolated EED was analyzed using generalized linear model adjusted for factors associated (*P*<0.1) with increased edema on univariate analysis. A *P*<0.05 in the multivariable models was considered significant. Propensity‐score matching was performed using *R* 3.1.0 MatchIt package while all other statistics were performed using SPSS 23 (IBM, Armonk, NY).

## Results

**Figure 1:**
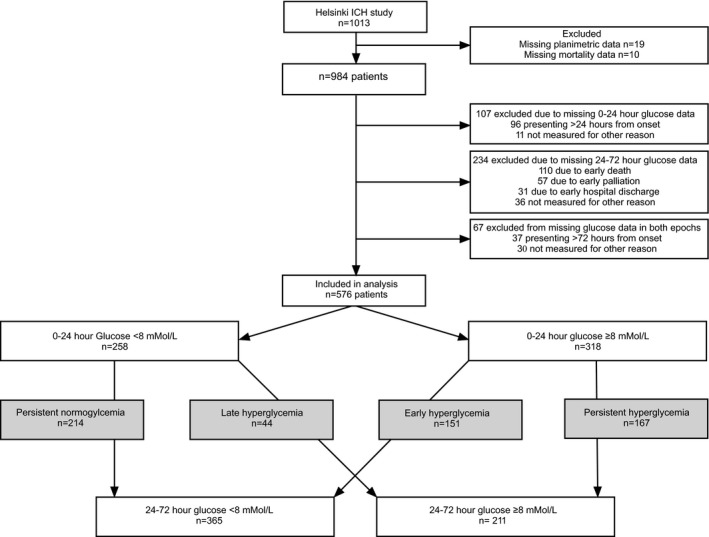
Study flow chart. ICH indicates intracerebral hemorrhage.

Of 1013 HICHS patients, 576 (57%) were eligible for the present analysis. Reasons for excluding 437 patients were the following: no planimetric data (n=19), missing 6‐month mortality (n=10), or missing glucose data (n=408) in either or both time windows mostly because of late presentation or early death/palliation, as illustrated in Figure 1. Excluded patients were older (70 versus 65), more likely to have a history of ischemic heart disease (15.8% versus 10.4%) or previous ICH (7.3% versus 3.8%), at baseline had lower GCS (14 versus 15), higher NIHSS (13 versus 10), larger ICH volume (18.0 mL versus 13.4 mL), absolute edema volume (14.4 mL versus 10.1 mL), and EED (0.37 cm versus 0.32 cm), more often irregular hematoma shape (55.4% versus 44.3%), infratentorial location (16.7% versus 12.0%), ventricular extension (45.1% versus 38.0%), and had higher 6‐month mortality (39.2% versus 23.8%). In the excluded patients because of early death or palliation (n=167), the highest recorded glucose in the 0 to 24‐hour epoch was higher than the included patients (9.40 mmol/L versus 8.30 mmol/L, *P*<0.001) and they were more likely to be hyperglycemic at 0 to 24 hours (123/167 [73.7%] versus 318/576 [55.2%], *P*<0.001). Excluded patients were thus either mild and late, or with characteristics making them prone to early palliation and death (Table S1).

### Baseline Patient Characteristics and Glycemic Trajectory

**Figure 2:**
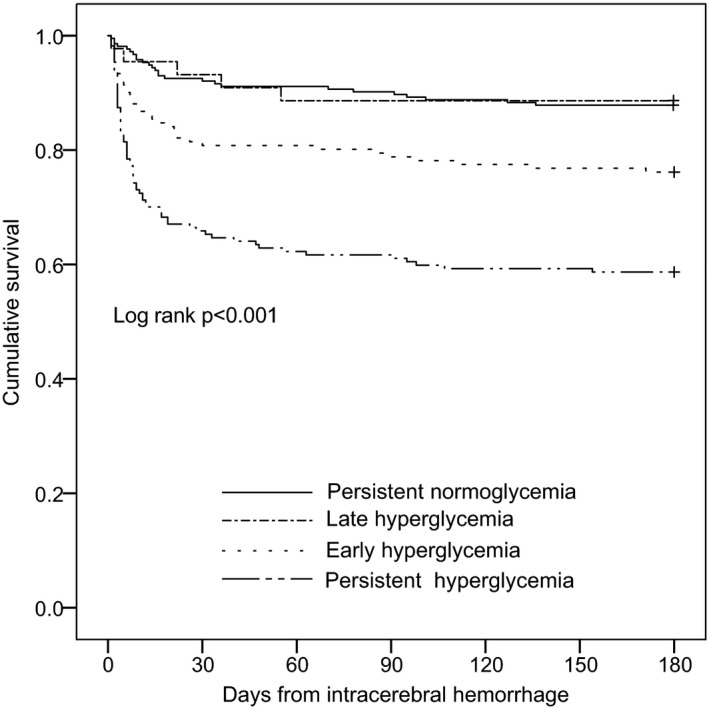
Kaplan–Meier survival curve according to glycemic trajectory.

Of the 576 patients in the analysis, 214 (37.2%) had persistent normoglycemia, 44 (7.6%) late only hyperglycemia, 151 (26.2%) early only hyperglycemia, and 167 (29.0%) persistent hyperglycemia. In 318 (55%) patients with hyperglycemia within 24 hours of ictus, 167 (53%) remained persistently hyperglycemic (Figure 1). The glycemic trajectory groups differed by presence of hypertension, diabetes mellitus, insulin, antiplatelet, anti‐hypertensive medication and statin use, rate of infection, neurosurgery, baseline NIHSS, GCS, ICH and absolute edema volumes, hematoma shape, infratentorial location, and ventricular extension (Table 1). The 6‐month mortality rate also differed between different glycemic trajectory groups, with the highest mortality observed in patients with early and persistent hyperglycemia on univariate analysis (Table 1, Figure 2).

**Table 1:** Baseline Patient Characteristics and Glycemic Trajectory

	Total, n=576	Persistent Normoglycemia, n=214	Late Hyperglycemia, n=44	Early Hyperglycemia, n=151	Persistent Hyperglycemia, n=167	*P* Value
Age	66 (57–84)	69 (58–86)	66 (57–84)	62 (57–85)	66 (58–84)	0.537
Male sex	342 (59.4%)	128 (59.8%)	24 (54.5%)	85 (56.3%)	105 (62.9%)	0.597
Hypertension	357 (62.0%)	109 (50.9%)	29 (65.9%)	89 (58.9%)	130 (77.8%)	<0.001
Diabetes mellitus	89 (15.5%)	5 (2.3%)	2 (4.5%)	14 (9.3%)	68 (40.7%)	<0.001
Atrial fibrillation	75 (13.0%)	27 (12.6%)	7 (15.9%)	20 (13.2%)	21 (12.6%)	0.942
Ischemic heart disease	60 (10.4%)	14 (6.5%)	6 (13.6%)	16 (10.6%)	24 (14.4%)	0.081
Cardiac failure^a^	30 (5.3%)	7 (3.3%)	2 (4.5)	12 (7.9%)	9 (5.5%)	0.268
Dyslipidemia^a^	121 (21.2%)	42 (19.7%)	12 (27.3%)	22 (14.8%)	45 (27.4%)	0.033
Previous ICH	22 (3.8%)	8 (3.7%)	3 (6.8%)	7 (4.6%)	4 (2.4%)	0.517
Antiplatelet use	138 (24.0%)	48 (22.4%)	6 (13.6%)	22 (14.6%)	62 (37.1%)	<0.001
Warfarin use	71 (12.3%)	25 (11.7%)	5 (11.4%)	22 (14.6%)	19 (11.4%	0.810
Anti‐hypertensive medication	267 (46.4%)	80 (37.4%)	19 (43.2%)	66 (43.7%)	102 (61.1%)	<0.001
Insulin use^a^	35 (6.3%)	1 (0.5%)	1 (2.3%)	2 (1.3%)	32 (19.3%)	<0.001
Statin use^a^	110 (19.3%)	37 (17.3%)	9 (20.5%)	16 (11.0%)	48 (28.9%)	0.001
Neurosurgery	64 (11.1%)	14 (6.5)	5 (11.4%)	28 (18.5%)	17 (10.2%)	0.004
Any infection^b^	305 (53.0%)	95 (44.4%)	24 (54.5%)	87 (57.6%)	99 (59.3%)	0.016
Baseline GCS	15 (12–15)	15 (14–15)	15 (13–15)	14 (10–15)	13 (10–15)	<0.001
Baseline NIHSS	10 (5–34)	7 (3–22)	8 (5–25)	13 (6–35)	13 (7–35)	<0.001
Time to baseline CT scan	2.7 (1.5–8.1)	3.0 (1.5–11.4)	4.4 (1.8–12.7)	2.3 (1.4–5.4)	2.5 (1.4–6.1)	0.006
Baseline ICH volume, mL	13.4 (5.6–79.2)	10.3 (3.9–57.3)	9.0 (4.3–73.2)	16.1 (7.2–85.3)	17.8 (7.8–96.0)	<0.001
Baseline edema volume, mL	10.1 (3.9–61.2)	9.0 (3.5–43.6)	10.6 (3.5–51.4)	10.9 (4.1–51.6)	10.3 (5.1–80.2)	0.047
Baseline EED, cm	0.32 (0.19–0.44)	0.33 (0.19–0.44)	0.33 (0.18–0.50)	0.30 (0.20–0.41)	0.31 (0.18–0.47)	0.733
72‐h EED, cm	0.87 (0.62–1.20)	0.60 (0.42–0.78)	0.60 (0.46–0.82)	0.65 (0.42–0.91)	0.63 (0.43–0.91)	0.429
Peak available EED, cm	0.51 (0.32–0.78)	0.51 (0.33–0.75)	0.51 (0.35–0.60)	0.51 (0.30–0.88)	0.54 (0.33–0.80)	0.496
Irregular hematoma shape^c^	255 (44.3%)	77 (36.0%)	15 (34.1%)	73 (48.7%)	90 (53.9%)	0.002
Infratentorial location	69 (12.0%)	18 (8.4%)	2 (4.5%)	28 (18.5%)	21 (12.6%)	0.011
Ventricular extension	219 (38.0%)	65 (30.4%)	11 (25.0%)	65 (43.0%)	78 (46.7%)	0.001
6‐mo mortality	137 (23.8%)	26 (12.1%)	5 (11.4%)	36 (23.8%)	70 (41.9%)	<0.001

Data are median (interquartile range) or n (%). CT indicates computed tomography; EED, edema extension distance; GCS, Glasgow Coma Scale; ICH, intracerebral hemorrhage; NIHSS, National Institutes of Health Stroke Scale.

a Missing data for prior statin (6) and insulin (3) use, history of cardiac failure (9), and dyslipidemia (6).

b Infection was defined as pneumonia, urinary tract infection, sepsis, or other infection treated with antibiotics.

c Hematoma shape not classified for 1 patient with pure IVH.

### Factors Associated With 6‐Month Mortality

There were 137 (23.8%) deaths by 6 months and factors associated with 6‐month mortality on univariate analysis were older age, prior use of warfarin or antihypertensive medication, evidence of infection, lower GCS, higher NIHSS, higher baseline ICH and absolute edema volumes, irregular hematoma shape, ventricular extension, and 72‐hour EED (Table 2).

**Table 2:** Baseline Patient Characteristics Associated With 6‐Month Mortality

	Total, n=576	Dead at 180 Days, n=137	Alive at 180 Days, n=439	*P* Value
Age, per y	66 (57–76)	72 (60–80)	65 (57–75)	<0.001
Male sex	342 (59.4%)	90 (65.7%)	252 (57.4%)	0.085
Hypertension	357 (62.0%)	93 (67.9%)	264 (60.1%)	0.103
Diabetes mellitus	89 (15.5%)	26 (19.0%)	63 (14.4%)	0.191
Atrial fibrillation	75 (13.0%)	22 (16.1%)	53 (12.1%)	0.226
Ischemic heart disease	60 (10.4%)	20 (14.6%)	40 (9.1%)	0.066
Cardiac failure^a^	30 (5.3%)	8 (6.0%)	22 (5.1%)	0.670
Dyslipidemia^a^	121 (21.2%)	24 (17.5%)	97 (22.2%)	0.305
Previous ICH	22 (3.8%)	6 (4.4%)	16 (3.6%)	0.695
Antiplatelet use	138 (24.0%)	34 (24.8%)	104 (23.7%)	0.787
Warfarin use	71 (12.3%)	24 (17.5%)	47 (10.7%)	0.034
Antihypertensive medication	267 (46.4%)	76 (55.5%)	191 (43.5%)	0.014
Insulin use^a^	36 (6.3%)	10 (7.5%)	26 (5.9%)	0.520
Statin use^a^	110 (19.3%)	26 (19.7%)	84 (19.2%)	0.895
Neurosurgery	64 (11.1%)	15 (10.9%)	49 (11.2%)	0.945
Any infection	305 (53.0%)	88 (64.2%)	217 (49.4%)	0.002
Baseline GCS	15 (12–15)	12 (7–14)	15 (13–15)	<0.001
Baseline NIHSS	10 (5–17)	18 (13–24)	8 (4–13)	<0.001
Time to baseline CT scan, h	2.7 (1.5–8.1)	2.4 (1.3–6.2)	2.9 (1.5–8.5)	0.087
Baseline ICH volume, mL	13.4 (5.6–34.2)	33.2 (13.5–62.0)	10.4 (4.3)	<0.001
Baseline edema volume, mL	10.1 (3.9–21.8)	18.3 (7.4–40.1)	9.0 (3.5–17.6)	<0.001
Baseline EED, cm	0.32 (0.19–0.44)	0.35 (0.20–0.48)	0.31 (0.18–0.44)	0.134
72‐h EED, cm	0.62 (0.43–0.86)	0.67 (0.49–0.92)	0.60 (0.41–0.85)	0.029
Irregular hematoma shape^b^	255 (44.3%)	93 (67.9%)	162 (37.0%)	<0.001
Infratentorial location	69 (12.0%)	18 (13.1%)	51 (11.6%)	0.632
Ventricular extension	219 (38.0%)	83 (60.6%)	136 (31.0%)	<0.001
Glycemic trajectory groups				<0.001
Persistent normoglycemia	214 (37.2%)	26 (19.0%)	188 (42.8%)	<0.001
Late hyperglycemia	44 (7.6%)	5 (3.6%)	39 (8.9%)	0.044
Early hyperglycemia	151 (26.2%)	36 (26.3%)	115 (26.2%)	0.985
Persistent hyperglycemia	167 (29.0%)	70 (51.1%)	97 (22.1%)	<0.001

Data are median (interquartile range) or n (%). CT indicates computed tomography; EED, edema extension distance; GCS, Glasgow Coma Scale; ICH, intracerebral hemorrhage; NIHSS, National Institutes of Health Stroke Scale.

a Missing data for prior statin (6) and insulin (3) use, history of cardiac failure (9), and dyslipidemia (6).

b Hematoma shape not classified for 1 patient with pure intraventricular hemorrhage.

In the multivariable logistic regression model, persistent hyperglycemia was associated with 6‐month mortality (odds ratio [OR] 3.675, 95% CI 1.989–6.792; *P*<0.001) adjusted for log‐transformed baseline ICH volume, baseline absolute edema volume, male sex, age, ventricular extension, infratentorial location, NIHSS, and GCS (Table 3). Based on the Wald statistic, glycemic trajectory had the strongest association with outcome of all the covariates. The variance inflation factor was <3.0 between the covariates in the model indicating no significant multicollinearity. The logistic regression model was of good fit (area under the curve 0.877, 95% CI 0.846–0.908) and the addition of glycemic trajectory into base models containing known predictors of outcome provided the best fit (Table S2).

**Table 3:** Multivariable Logistic Regression Model on Factors Associated With 6‐Month Mortality

	All Patients, n=576			Excluding Diabetic Patients, n=487		
	OR	*P* Value	Wald	OR	*P* Value	Wald
Glycemic trajectories	···	<0.001	22.288	···	<0.001	24.882
Late hyperglycemia^a^	0.965 (0.298–3.129)	0.952	0.004	1.046 (0.324–3.380)	0.940	0.006
Early hyperglycemia^a^	1.290 (0.657–2.535)	0.459	0.547	1.350 (0.669–2.724)	0.402	0.702
Persistent hyperglycemia^a^	3.675 (1.989–6.792)	<0.001	17.252	5.139 (2.545–10.376)	<0.001	20.843
Log of baseline ICH volume, per 1^b^	3.515 (1.493–8.276)	0.004	8.274	3.724 (1.437–9.651)	0.007	7.326
Baseline edema volume, mL	0.997 (0.982–1.012)	0.727	0.122	0.994 (0.977–1.011)	0.462	0.542
Age, per y	1.054 (1.030–1.078)	<0.001	20.664	1.058 (1.032–1.085)	<0.001	19.834
Male sex	1.719 (1.033–2.861)	0.037	4.349	1.700 (0.971–2.975)	0.063	3.448
Warfarin use	1.981 (0.997–3.935)	0.051	3.807	2.761 (1.249–6.104)	0.012	6.298
Baseline NIHSS, per point	1.097 (1.051–1.145)	<0.001	18.175	1.096 (1.043–1.151)	<0.001	13.204
Baseline GCS, per point	0.954 (0.863–1.055)	0.362	0.831	0.964 (0.857–1.084)	0.540	0.376
Infratentorial location	1.208 (0.533–2.739)	0.651	0.205	1.242 (0.518–2.977)	0.627	0.236
Ventricular extension	1.746 (1.060–2.877)	0.029	4.783	1.628 (0.929–2.852)	0.088	2.902

GCS indicates Glasgow Coma Scale; ICH, intracerebral hemorrhage; NIHSS, National Institutes of Health Stroke Scale; OR, odds ratio.

a Compared with persistent normoglycemia.

b All baseline ICH volume had addition of 1.0 before log transformation to allow inclusion of 3 patients with 0 baseline volume because of pure ventricular hemorrhage.

Neither surgery (OR 0.560, 95% CI 0.247–1.270, *P*=0.165) nor presence of infection (OR 0.911, 95% CI 0.546–1.540, *P*=0.744) was associated with 6‐month mortality when included in the model or influenced the association of persistent hyperglycemia and mortality (with surgery—OR 3.676, 95% CI 1.985–6.807, *P*<0.001; with infection—OR 3.693, 95% CI 1.997–6.828, *P*<0.001.) There was also no interaction of surgery (*P*=0.965) or presence of infection (*P*=0.594) on the association between glycemic trajectory and mortality. The association with 6‐month mortality in the persistent hyperglycemia group remained significant after excluding the 89 patients with diabetes mellitus (OR 5.139, 95% CI 2.545–10.376, *P*<0.001).

In the secondary analyses, glucose as a continuous variable was associated with 6‐month mortality in both the 0 to 24‐hour (OR 1.075 per mmol/L increase, 95% CI 1.018–1.135, *P*=0.009) and 24 to 72‐hour (OR 1.140 per mmol/L increase, 95% CI 1.050–1.238, *P*=0.002) epochs. There was a significant correlation between the maximum glucose measurements in each epoch (Pearson correlation 0.541, *P*<0.001). Absolute glucose change between the maximum readings from the 2 epochs was also associated with 6‐month mortality (OR 1.101 per mmol/L increase, 95% CI 1.002–1.210, *P*=0.046) in the logistic regression analysis adjusting for the predefined covariates and maximum glucose from 0 to 24‐hour epoch. In the 184 (31.9%) patients with baseline (<12 hours from ictus) and follow‐up computed tomography (12–72 hours from ictus) available, introducing hematoma growth into the logistic regression model did not influence the association of persistent hyperglycemia on mortality (OR 3.674, 95% CI 1.307–10.332, *P*=0.014).

### Propensity‐Score Matching Analysis

Propensity‐score matching resulted in a reduced number of patients in each glycemic trajectory group (total n=266; glycemic trajectory groups: persistent normoglycemia n=81, early only hyperglycemia n=71, late only hyperglycemia n=44, and persistent hyperglycemia n=70) with matched baseline characteristics (Table S3). In the multivariable logistic regression analysis, the association between persistent hyperglycemia and mortality remained (OR 3.653, 95% CI 1.357–9.836, *P*=0.010) (Table S4).

### Association Between Glycemic Trajectory and Interpolated 72‐Hour EED

In the generalized linear model analyses, there was no association between glycemic trajectory groups and interpolated 72‐hour EED in either univariate (*P*=0.426) or multivariable analyses (*P*=0.493, Table S5, adjusted for log baseline ICH volume, diabetes mellitus, history of hypertension, irregular hematoma shape, baseline NIHSS, ventricular extension, and infratentorial location).

## Discussion

We have shown in a large sample of ICH patients that early hyperglycemia is only associated with ICH mortality if the hyperglycemia is persistent. The association of persistent hyperglycemia with mortality remained robust even after excluding patients known to have diabetes mellitus and following propensity‐score‐matching analysis.

A large number of observational studies (21 studies, total number of patients=12 145, Table S6)^2^, ^3^, ^4^, ^5^, ^6^, ^7^, ^8^, ^9^, ^10^, ^11^, ^22^, ^23^, ^24^, ^25^, ^26^, ^27^, ^28^, ^29^, ^30^, ^31^, ^32^ have examined the association between glucose with ICH outcome with the majority (15 studies, total n=11 161) defining hyperglycemia on the basis of single glucose measurement. One notable study was the post hoc analysis of the Intensive Blood Pressure Reduction in Acute Cerebral Hemorrhage Trial (INTERACT) II study (n=2653).^30^ The authors assessed the prognostic significance of admission glucose level on 90‐day outcome. This study reported that admission glucose both as a continuous variable and also the highest quartile (>7.9 mmol/L) was associated with combined outcome of death or disability (adjusted OR per mmol/L glucose 1.35, 95% CI 1.01–1.33, *P*=0.043; adjusted OR for fourth quartile of glucose level 1.34, 95% CI 1.01–1.80, *P* for trend 0.015).

Six studies (n=984)^6^, ^7^, ^8^, ^9^, ^10^, ^11^ reported results for more than single glucose measurement. Although studies by Tapia‐Perez et al^10^ and Koga et al^11^ reported an association between glucose and outcome (Table S6), these studies (n=298) derived the outcome association from a single glucose measurement, even though multiple time points were reported. Our secondary analyses using glucose as continuous variable at both the 0 to 24 and 24 to 72‐hour epochs are in agreement with these findings. However, we can only report the true natural history of glucose below the cutoff for glucose‐lowering treatment and our results do not provide insight into the natural peak of glucose reached in hyperglycemic patients. The results of the secondary analyses therefore need to be interpreted within this context.

Four studies (Table S6) evaluated glycemic trajectory on ICH outcome. In the post hoc analysis of the Antihypertensive Treatment of Acute Cerebral Hemorrhage I (ATACH I, n=60)^8^ study, increasing glycemic trend in the first 72 hours was associated with 2.5‐fold increase in risk of 90‐day death or disability on univariate analysis, but this was not significant when adjusted for GCS, ICH volume, and ventricular extension (relative risk 1.19, 95% CI 0.92–1.54, *P* value not reported). Feng et al assessed the impact of hyperglycemia (mean glucose over 72 hours of ≥150 mg/dL) in 135 ICH patients^9^ and found no association between hyperglycemia and 90‐day death or disability (OR 1.06, 95% CI 0.42–2.66, *P* value not reported). Godoy et al reported in 295 patients that those with persistent hyperglycemia in the 72 hours after ICH (n=78) had higher 30‐day mortality (80%) compared with other glycemic trajectory groups on univariate analysis (*P*<0.001), but no multivariable analysis was reported.^7^ Schwarz et al analyzed the impact of persistent hyperthermia at 72 hours on poor outcome (OR for hyperthermia >48‐hour duration 13.52, 95% CI 2.22–82.23, *P*=0.005) in 196 ICH patients.^6^ In the multivariable analysis, persistent hyperglycemia (defined as >11.0 mmol/L for more than 24‐hour duration, n=32) was associated with poor discharge outcome (OR 13.54, 95% CI 2.24–81.78, *P*=0.005). There was no association between hyperglycemia of <24‐hour duration with outcome (Table S6). Our results, derived from a significantly larger sample size (n=576), are in concordance with that reported by Schwarz,^6^ and suggests that hyperglycemia is only harmful after ICH if it persists.

We did not find a significant association with ICH mortality in the subgroup of patients (n=44, 7.7% of cohort) classified with late‐only hyperglycemia (OR 0.965, 95% CI 0.298–3.129, *P*=0.952). These results were derived from a small sample size with a point estimate that approached 1 and a wide confidence interval. The negative association needs to be considered in this context and late hyperglycemia should still be managed as per current best practice guidelines.^1^ Although our secondary analyses showed increasing glucose levels in both epochs to be associated with mortality, it is likely the outcome association is driven by patients with persistent rather than transient hyperglycemia as demonstrated in the primary analysis.

The mechanism through which hyperglycemia mediates ICH outcome is uncertain. It is plausible that hyperglycemia reflects more severe brain injury resulting from larger baseline hematoma volume (Table 1). However, our logistic regression analyses indicate the robust association of persistent hyperglycemia and mortality even after adjusting for surgery, evidence of infection, and hematoma growth, which are factors that also influence outcome. It is possible that hyperglycemia exacerbates secondary injury. In rat ICH models, hyperglycemia was associated with increased neuronal death and brain edema caused by worsened blood–brain barrier disruption.^12^, ^13^ However, data from INTERACT II showed no association between baseline hyperglycemia and 24‐hour absolute edema growth (glucose ≥6.5 mmol/L versus <6.5 mmol/L, 2.6 mL versus 3.0 mL edema growth, respectively, *P*=0.293).^30^ We were also unable to demonstrate an association between hyperglycemia and 72‐hour EED. In humans, hyperglycemia induces inflammatory cytokines and the effect persists until return to normoglycemic state.^33^ Further, there is evidence of progressive increase in perihematomal glucose metabolism in human ICH peaking at day 3, likely in response to increased perihematomal inflammatory cell infiltrate.^34^ It is therefore likely that hyperglycemia contributes to secondary neuronal injury by exacerbating the cerebral inflammatory milieu and oxidative stress,^35^ resulting in cellular injury in a process that is difficult to quantify clinically. The effects of hyperglycemia on brain injury are additive to primary injury resulting from the hematoma. Finally, hyperglycemia is also associated with increased risk of cardiac and infectious complications.^2^

Two large randomized controlled trials have examined glycemic control on stroke outcome. The UK Glucose Insulin in Stroke Trial (GIST‐UK) enrolled 933 (134 [14.4%] were ICH) stroke patients with admission glucose between 6.0 and 17.0 mmol/L to targeted glycemic control (4.0–7.0 mmol/L) or no intervention.^36^ There was no reduction in 90‐day mortality in the intervention group (OR 1.14, 95% CI 0.86–1.51, *P*=0.37). The Quality in Acute Stroke Care (QASC) study randomized 1126 (51 [4.5%] were ICH) acute stroke patients in stroke units to a set of protocolized interventions for managing glucose, fever, and swallowing dysfunction or guideline management. The intervention group had reduced likelihood of 90‐day death or disability (42% versus 58%, *P*=0.002).^37^ Interpreting these findings in ICH is difficult because of the small number of patients. In QASC it is unclear which of the 3 interventions contributed to mortality reduction.^37^ In GIST‐UK >50% of patients had baseline glucose of <8.0 mmol/L, below the threshold for insulin treatment used in the present study. Therefore, our results cannot be interpreted in the light of intervention because only the persistent normoglycemia group (n=214, 37%) received no insulin treatment and in the remaining 362 (63%) patients the glucose was spontaneously elevated before treatment. Although our results are based on a retrospective cohort, the consistent association between hyperglycemia in this study and previous observational data indicate an urgent need to assess glycemic management on ICH outcome in randomized controlled trials.

We acknowledge the study limitations including firstly the potential for bias and chance associations in retrospective studies. We tried to minimize bias by predefining our study population and analysis a priori. Secondly, we had to exclude 43% of the patients in the HICHS predominately because of early death, early palliation, or late presentation (Figure 1). The excluded patients had worse neurological injury and higher mortality (Table S1), thus the clinical relevance of hyperglycemia in these patients is less clear. Thirdly, we do not have hemoglobin A1C measurements in most patients, and some patients with persistent hyperglycemia may have undiagnosed diabetes mellitus. Although diabetic patients had more persistent hyperglycemia, diabetes mellitus was not associated with mortality in the present cohort. Furthermore, the association of persistent hyperglycemia with mortality remained after excluding patients with known diabetes mellitus and also after propensity‐score‐matching analysis. Fourthly, the lack of association between glycemic trajectory and 72‐hour edema was based on EED derived from interpolating EED volume obtained at a median time of 24 hours from ictus. The EED may not accurately represent the natural evolution in these patients, and our negative association needs to be interpreted in the context of this limitation. Fifth, we do not have information on functional outcome or medical causes of death and were unable to provide insight into potential associations with hyperglycemia. Finally, the results are derived from a single‐center study, which may limit generalizability.

## Conclusion

Over half of ICH patients experienced early hyperglycemia, which is only associated with higher mortality when it persists. Strategies to achieve glycemic control after ICH may influence patient outcome and need to be assessed in randomized controlled trials.

## Sources of Funding

Wu is supported by grants from the Neurological Foundation of New Zealand (grant number 1313‐CF) and Royal Melbourne Hospital Neuroscience Foundation; Strbian is supported by grants from the Helsinki University Central Hospital and the Finnish Medical Foundation; Tatlisumak is supported by the Helsinki University Central Hospital and Sahlgrenska University Hospital grants for ICH research; Meretoja is supported by grants from National Health and Medical Research Council (Australia), Academy of Finland, and the Finnish Medical Foundation.

## Disclosures

None.

## Supporting information


**Table S1.** Baseline Characteristics Between Included and Excluded Patients
**Table S2.** Model Fit With Different Logistic Regression Models
**Table S3.** Baseline Characteristics in the Propensity‐Score‐Matched Population
**Table S4.** Multivariable Logistic Regression Model in Propensity‐Score‐Matched Population on Factors Associated With 6‐Month Mortality
**Table S5.** Generalized Linear Model on Association of Glycemic Status on Extrapolated 72‐Hour Edema Extension Distance (EED) in Centimeters
**Table S6.** Studies Investigating the Association of Glucose and Outcome After Intracerebral Hemorrhage
