# Supplementary material for: Persistent Hyperglycemia Is Associated With Increased Mortality After Intracerebral Hemorrhage
Source: J Am Heart Assoc. 2017 Aug 2;6(8):e005760. doi: 10.1161/JAHA.117.005760 (PMC5586431; doi:10.1161/JAHA.117.005760)
Supplement: Supplementary file 1 — Table S1. Baseline Characteristics Between Included and Excluded Patients Table S2. Model Fit With Different Logistic Regression Models Table S3. Baseline Characteristics in the Propensity‐Score‐Matched Population Table S4. Multivariable Logistic Regression Model in Propensity‐Score‐Matched Population on Factors Associated With 6‐Month Mortality Table S5. Generalized Linear Model on Association of Glycemic Status on Extrapolated 72‐Hour Edema Extension Distance (EED) in Centimeters Table S6. Studies Investigating the Association of Glucose and Outcome After Intracerebral Hemorrhage [file JAH3-6-e005760-s001.pdf]

SUPPLEMENTAL MATERIAL

Table S1. Baseline characteristics between included and excluded patients.

	Total n=1013	Included n=576	Excluded n=437	p
Age	68 (58-78)	66 (57-76)	70 (59-79)	<0.001
Male sex	582 (57.5%)	342 (59.4%)	240 (54.9%)	0.159
Hypertension	637 (62.9%)	357 (62%)	280 (64.1%)	0.512
Diabetes mellitus	148 (14.6%)	89 (15.5%)	59 (13.5%)	0.419
Atrial fibrillation	146 (14.4%)	75 (13.0%)	71 (16.2%)	0.150
Ischemic heart disease	129 (12.7%)	60 (10.4%)	69 (15.8%)	0.013
Cardiac failure	49 (4.9%)	30 (5.3%)	19 (4.4%)	0.558
Dyslipidemia	197 (19.7%)	121 (21.2%)	76 (17.7%)	0.173
Previous ICH	54 (5.3%)	22 (3.8%)	32 (7.3%)	0.016
Antiplatelet use	265 (26.2%)	138 (24.0%)	127 (29.1%)	0.071
Warfarin use	133 (13.1%)	71 (12.3%)	62 (14.2%)	0.399
Anti-hypertensive medication	489 (48.3%)	267 (46.4%)	222 (50.8%)	0.163
Insulin use	54 (5.4%)	36 (6.3%)	18 (4.2%)	0.160
Statin use	191 (19.2%)	110 (19.3%)	81 (19.0%)	0.935
Baseline GCS	14 (10-15)	15 (12-15)	14 (8-15)	<0.001
Baseline NIHSS	11 (4-20)	10 (5-17)	13 (4-25)	0.008
Time to baseline CT scan, hours	3.8 (1.6-16.0)	2.7 (1.5-8.1)	9.8 (2.0-43.0)	<0.001

Baseline ICH volume	14.25 (5.7-40.1)	13.4 (5.6-34.2)	18.0 (6.1-52.2)	0.002
Baseline edema volume	11.35 (4.1-27.7)	10.1 (3.9-21.8)	14.4 (4.6-37.5)	<0.001
Baseline EED, per cm	0.34 (0.19-0.48)	0.32 (0.19-0.44)	0.37 (0.21-0.52)	<0.001
Peak EED, per cm	0.48 (0.28-0.71)	0.51 (0.32-0.78)	0.42 (0.22-0.64)	<0.001
72 hour EED, per cm	0.60 (0.29-0.86)	0.62 (0.43-0.86)	0.56 (0.36-0.86)	0.021
Irregular hematoma shape	484 (49.0%)	255 (44.3%)	229 (55.4%)	0.001
Infratentorial location	142 (14.0%)	69 (12.0%)	73 (16.7%)	0.036
Ventricular extension	416 (41.1%)	219 (38.0%)	197 (45.1%)	0.024
6-month mortality	347 (34.6%)	137 (23.8%)	210 (39.2%)	<0.001

Data are median (interquartile range) or n (%).
Abbreviations: EED, edema extension distance; GCS, Glasgow Coma Scale; ICH, intracerebral hemorrhage; NIHSS, National Institutes of Health Stroke Scale

Table S2. Model fit with different logistic regression models.

Variables in model	AUC
Log baseline ICH volume and age	0.761 (0.717-0.808)
All predefined variables excluding glycemic trajectory	0.863 (0.829-0.897)
Full model*	0.877 (0.846-0.908)

Abbreviations: AUC, area under curve; ICH, intracerebral hemorrhage.
*Covariates in the full model are log baseline ICH volume, baseline National Institutes of Health Stroke Scale, baseline Glasgow Coma Scale score, baseline edema volume, age, previous warfarin use, infratentorial location and presence of ventricular extension.

Table S3. Baseline characteristics in the propensity score matched population.

	Total n=266	Persistent normoglycemia n=81	Late hyperglycemia n=44	Early hyperglycemia n=71	Persistent hyperglycemia n=70	p
Age	66 (57-76)	69 (57-76)	66 (57-77)	62 (57-77)	65 (58-76)	0.800
Male sex	144 (54.1%)	40 (49.4%)	24 (54.5%)	40 (56.3%)	40 (57.1%)	0.769
Warfarin use	31 (11.7%)	8 (9.9%)	5 (11.4%)	9 (12.7%)	9 (12.9%)	0.936
Baseline GCS	15 (13-15)	15 (14-15)	14 (13-15)	15 (12-15)	15 (13-15)	0.574
Baseline NIHSS	9 (4-15)	6 (3-14)	8 (5-13)	10 (6-16)	12 (6-16)	0.066
ICH volume	12.6 (5.7-27.8)	11.8 (5.8-26.3)	9.0 (4.3-24.4)	15.7 (5.9-33.8)	13.1 (6.2-26.9)	0.363
Baseline edema volume, mL	10.8 (4.1-21.3)	11.9 (5.2-21.0)	10.6 (3.5-15.2)	12.2 (4.1-24.2)	7.6 (3.9-20.5)	0.318
Infratentorial location	11 (4.1%)	2 (2.5%)	2 (4.5%)	3 (4.2%)	4 (5.7%)	0.795
Ventricular extension	75 (28.2%)	25 (30.9%)	11 (25.0%)	20 (28.2%)	19 (27.1%)	0.909
6-month mortality	45 (16.9%)	10 (12.3%)	5 (11.4%)	8 (11.3%)	22 (31.4%)	0.003

Data are median (interquartile range) or n (%).
GCS, Glasgow Coma Scale; ICH, intracerebral hemorrhage; NIHSS, National Institutes of Health Stroke Scale.

Table S4. Multivariable logistic regression model in propensity score matched population on factors associated with 6-month mortality.

	All patients, n=266	
	OR	p
Glycemic trajectories	-	0.004
Late hyperglycemia*	0.928 (0.250-3.448)	0.911
Early hyperglycemia*	0.608 (0.190-1.944)	0.402
Persistent hyperglycemia*	3.653 (1.357-9.836)	0.010
Log of baseline ICH volume, per 1†	4.000 (0.889-17.991)	0.071
Baseline edema volume, mL	0.997 (0.972-1.022)	0.809
Age, per year	1.057 (1.019-1.095)	0.003
Male sex	2.029 (0.887-4.643)	0.094
Warfarin use	2.955 (1.045-8.350)	0.041
Baseline NIHSS, per point	1.126 (1.041-1.218)	0.003
Baseline GCS, per point	1.025 (0.849-2.243)	0.796
Ventricular extension	0.933 (0.388-2.243)	0.877
Infratentorial location ‡	-	-

*Compared with persistent normoglycemia; GCS indicates Glasgow Coma Scale; ICH, intracerebral hemorrhage; NIHSS, National Institutes of Health Stroke Scale. †All baseline ICH volume had addition of 1.0 prior to log transformation to allow inclusion of 3 patients with 0 baseline volume due to pure ventricular hemorrhage. ‡ No deaths occurred in patients with infratentorial hemorrhage in the propensity score matched population.

Table S5. Generalized linear model on association of glycemic status on extrapolated 72-hour edema extension distance (EED) in centimeters.

	beta	SE	Wald	p
Glycemic trajectory	-	-	2.401	0.493
Normoglycemia (reference)	-	-	-	-
Late hyperglycemia	-0.004	0.092	0.002	0.965
Early hyperglycemia	0.091	0.062	2.203	0.138
Persistent hyperlycemia	0.050	0.067	0.561	0.454
Log ICH vol, per 1	0.334	0.066	25.781	<0.001
Irregular hematoma shape	0.055	-0.057	0.938	0.333
Ventricular extension	-0.184	0.051	13.243	<0.001
Infratentorial location	-0.474	0.074	41.101	<0.001
NIHSS, per point	0.005	0.003	2.893	0.089
Hypertension	-0.033	0.502	0.435	0.510
Diabetes mellitus	-0.205	0.074	7.787	0.005

ICH indicates intracerebral hemorrhage; NIHSS, National Institutes of Health Stroke Scale; SE, standard error

Table S6. Studies investigating the association of glucose and outcome after intracerebral hemorrhage.

**Studies that used baseline blood glucose only (15 studies, n=11,161)**							
Author, year	Country	Study size	Proportion diabetic	Timing of glucose measurement	Outcome measures	Main findings	Other adjusted covariates in multivariable analysis
Franke[1] 1992	Netherlands	157	NR	On admission, 96% presented within 24 hours of onset	2 day and 1 year mortality	Hyperglycemia (≥8 mMol/L) was associated with mortality (OR 5.5, P<0.001) at 2 days but not at 1 year	Age, hypertension, eye and motor scores on GCS, ICH volume, pineal gland displacement, modified Rankin score 5
Passero[2] 2003	Italy	739	127 (17%)	On admission, all patients presented within 24 hours of onset	30 and 90 day mortality	Hyperglycemia (≥130mg/dL / 7.22 mMol/L) in non-diabetic, non-comatose patients (n=415) was associated with mortality at 30 days (OR 1.290 95% CI 1.054-1.578, p=0.013) and at 90 days (1.269 (1.051-1.532, p=0.013)	Age, ICH volume, IVH, GCS, mean arterial pressure, surgical evacuation.
Fogelholm[3] 2005	Finland	329	39 (11.9%)	On admission, 89% presented within 24 hours of onset	28 day mortality	Increasing admission glucose (OR 1.22 per mMol/L 95% CI 1.07-1.40, p=0.004) was independently associated with 28-day mortality	Coma, midline shift, anticoagulant use, mean arterial pressure
Kimura[4] 2007	Japan	100	NR	On admission prior to CT scan. Mean time from onset to scan was 4.6 +/- 5.0 hours	14 day mortality	Hyperglycemia (>150mg/dL / 8.4 mMol/L) was associated with 14 day mortality (OR 35.34, 95% CI 1.40-992.73, p=0.031)	Age >70, systolic blood pressure, leucocytes >8.5, erythrocytes <0.37, potassium <3.5 mMol/L, albumin <4.0g/dL, Sodium <140 mMol/L, ICH volume >20 mL.
Tetri[5] 2009	Finland	379	68 (17.9%)	On admission, 94.2% presented within 48 hours of onset	2 and 90 day mortality	Admission glucose was not associated with 2 day (RR 1.04 (95% CI 0.95-1.13, p value not reported) or 90 day mortality (RR 1.04 95% CI 0.99-1.10, p value not reported)	Mean arterial pressure, sex, ICH volume, IVH, age, GCS, basal ganglia location, thalamus location, infratentorial location, cardiac disease, warfarin use.
Lee[6] 2010	South Korea	1387	161 (11.6%)	Fasting morning glucose the day after admission. All patients presented within 48 hours of onset	30 day and mortality on last follow up	Increasing glucose (HR 1.10 per mMol/L 95% CI 1.01-1.19, p=0.03) was associate with 30 day mortality but not long term mortality (HR 1.05 (0.98-1.11, p=0.15) Highest quartile (>9.30 mMol/L, HR 3.34 95% CI 1.15-0.73*, p=0.03) was associated with 30 day mortality but not long term mortality (HR 1.45 95% CI 0.83-2.53, p=0.19)	Age, diabetes, systolic and diastolic blood pressure, pontine location, ICH volume, IVH, GCS
Stead[7] 2010	USA	237	47 (19.8%)	On admission. Time of presentation in relation to symptom onset was not reported	7 day mortality	Hyperglycemia (>140mg/dL / 7.7mmol/L) was associated with 7-day mortality in non-diabetic patients (estimate 2.796 SE 1.174, p=0.0172) but not in diabetic patients (estimate 2.590 SE 2.197, p=0.2384)	Yes<br>Age, ICH volume, IVH, NIHSS.
Samiullah[8] 2010	Pakistan	399	NR	On admission and at 72 hours. All	In-hospital mortality	Hyperglycemia (fasting > 126 mg/dL / 7 mMol/L on admission or fasting glucose, or random glucose	Age, ICH volume, GCS

				patients presented within 24 hours of onset		>200mg/dL/11.1 mMol/L on 2 occasions) was associated with in-hospital mortality (OR 10.9 95% CI 4.72-25.32, p<0.001). However, the proportion of hyperglycemia and duration determined by random glucose testing was not reported.	
Di Napoli[9] 2011	Italy	210	58 (27.6%)	On admission. All patients presented within 24 hours of onset	30 day mortality	No association of highest quartile of glucose (>10.8 mMol/L / 194mg/dL) and mortality<br>Model 1: OR 7.16 95% CI 0.65-78.35, p=0.1067<br>Model 2: OR 10.39 95% CI 0.74-146.76, p=0.0832<br>Model 3: OR 9.38 95% CI 0.75-117.28, p=0.0824	Model 1: ICH volume, IVH, infratentorial location, age, time to blood test<br>Model 2: variables in model 1 plus mid line shift, hydrocephalus.<br>Model 3: variables in model 2 and surgery
Appelboom[10] 2011	USA	104	26 (23.6%)	On admission. Time of presentation in relation to symptom onset was not reported	Mortality on discharge and at 90 days	Critical hyperglycemia (>10mMol/L) was independently associated with mortality on discharge (OR 4.381 95% CI 1.186-16.174, p=0.009) and at 90 day (OR 10.85 95% CI 1.886-62.41, p=0.011)	Age, female sex, diabetes mellitus, GCS, AVM, ICH volume, IVH score, midline shift, infratentorial location, ventricular drain, intrathecal tPA, ventriculoperitoneal shunt, early DNR status, length of hospital stay.
Wang[11] 2011	China	189	31 (16.4%)	Within 24 hours of hospitalization. Time of presentation in relation to symptom onset not reported	30 day Barthel's Index	Hyperglycemia (>6.8 mMol/L) in non-diabetic patients (OR 0.081 95% CI 0.039-0.167, p<0.0001) and diabetic patients (OR 0.056 95% CI 0.022-0.142, p<0.0001) was associated with poor functional recovery.	ICH location, ICH volume and complications.
Bejot[12] 2012	France	419	68 (14.8%)	On admission. Time of presentation in relation to symptom onset not reported	1 month mortality	Hyperglycemia (≥8.6 mMol/L) was associated with 1 month mortality. (HR 1.76 95% CI 1.23-2.53, p=0.002)	Age, sex, ICH location, IVH, smoking status, aphasia, motor deficit, anticoagulant use, altered consciousness
Saxena[13] 2016	INTERACT II	2653	292 (11.0%)	On admission, all patients presented within 6 hours of onset.	90 day death, death or major disability (modified Rankin Score 3-6)	Glucose (per mMol/L) was associated with death (OR 1.16 95% CI 1.01-1.33, p=0.043), death or major disability (OR 1.11 95% CI 1.00-1.24, p<0.0001).<br><br>Highest quartile of glucose (7.9-25mMol/L) was associated with death or major disability (OR 1.35 95% CI 1.01-1.80, p trend 0.015).	Age, geographic region, sex, heart disease, hypertension, diabetes mellitus, use of aspirin or warfarin, ICH volume, ICH location, IVH, systolic blood pressure, randomized treatment, NIHSS ≥15, age x NIHSS ≥15 interaction, China x IVH interaction, ICH volume deep ICH location interaction and deep ICH x IVH interaction.
Liu[14] 2016	China	908	58 (6.8%)	On admission. Time of presentation in relation to	Death or disability (modified Rankin Score 3-6) at 3 and 12 months	Baseline glucose (per mMol/L) was associated with reduced odds of good outcome at 3 months (OR 0.914 95% CI 0.857-0.974, p=0.006) but not 12 months (point estimate not reported).	NIHSS, GCS, hematocrit, blood urea nitrogen, previous stroke, stroke complications

				symptom onset was not reported			
Sun[15] 2016	China	2951	267 (9.0%)	On admission. All patients presented within 24 hours of onset	Death or disability (modified Rankin Scale score 3-6) at 3 months	Glucose per mMol/L was associated with death or disability at 3 months (aOR 1.09 95% CI 1.04-1.15, p<0.001).<br><br>Highest quartile of glucose (≥7.53 mMol/L) was associated with poor 3-month outcome (aOR 1.54 95% CI 1.17-2.03, p=0.002).	Age, sex, hypertension, cardiovascular disease, atrial fibrillation, smoking, baseline ICH volume, ICH location, IVH, premorbid modified Rankin Scale score, NIHSS, GCS, admitted department, in-hospital treatment with dehydration agent, craniotomy and withdrawal of support.

Table S6 (continued)

**Studies that used serial glucose measurements (2 studies, n=298)**							
Author, year	Country	Study size	Proportion diabetic	Timing of glucose measurement	Outcome measures	Main findings	Other adjusted covariates in multivariable analysis
Tapia-Perez[16] 2014	Germany	122	37 (30.3%)	On admission, day 1 and 3. All patients presented within 24 hours of symptoms onset	Mortality at day 7 and 30	Hyperglycemia (>140mg/dL / 7.78 mMol/L) on day 1 was associated with 30-day mortality (HR 2.65 95% CI 1.15-6.12, p=0.02). No association with 7 day mortality was observed (point estimate not reported)	IVH, hydrocephalus, ICH volume, WCC, ventricular drain, age and GCS
Koga[17] 2015	Japan	176	22 (12.5%)	On admission, 24 and 72 hour. All patients presented within 3 hours of symptom onset	3 month outcomes – none to minimal disability, bedridden/death	Admission glucose (per 10mg/dL) was not associated with either none/minimal disability (OR 0.90 95% CI 0.77-1.02 p=0.099) or bedridden/death (OR 1.01 95% CI 0.84-1.19 p=0.892).<br>24 hour glucose (per 10mg/dL) was associated with none/minimal disability (OR 0.85 95% CI 0.69-0.98 p=0.021) and with bedridden/death (OR 1.14 95% CI 1.00-1.30 p=0.049)<br>72 hour glucose was associated with none/minimal disability (0.75 95% CI 0.59-0.92 p=0.003) but not with bedridden/death (OR 1.11 95% CI 0.98-1.27 p=0.101)	Sex, age, antithrombotic medication use, systolic blood pressure, initial heart rate, NIHSS, time to initial scan, ICH volume

Table S6 (continued)

Studies that used glucose trajectory (4 studies, n=686)							
Author, year	Country	Study size	Proportion diabetic	Timing of glucose measurement	Outcome measures	Main findings	Other adjusted covariates in multivariable analysis
Schwarz[18] 2000	Germany	196	NR	Admission to 72 hours. All patients presented within 24 hours of symptom onset	Discharge Glasgow Outcome Scale	Persistent hyperglycemia (>11.mMol/L) of >24 hour duration was associated with discharge outcome (OR 13.54 95% CI 2.24-81.78, p=0.005). Hyperglycemia less than 24 hours duration was not associated with discharge outcome (OR 1.72 95% CI 0.42-6.96, p=0.45)	IVH, GCS, ICH volume, ICH growth or new ICH, persistent hypertensive, persistent hyperthermia.
Godoy[19] 2008	Argentina, Italy	295	148 (50.2%)	Daily blood glucose for 72 hours. All patients presented within 24 hours of symptom onset.	30 day mortality	On univariate analysis mortality was highest in patients with persistent hyperglycemia (80%) compared to those with increasing glucose pattern (40%), decreasing glucose pattern (36%) and persistently normal (9%), p<0.001.<br>Glucose level (per mMol/L) between 0-12 hours (OR 1.33 95% CI 1.19-1.50, p<0.0001) and 49-72 hours (OR 1.38 95% CI 1.16-1.65 p<0.0001) was independently associated with 30 day mortality.<br>Glucose level between 13-24 hours (OR 1.12 OR 0.98-1.28 p=0.0861) and 25-48 hours (OR 1.09 95% CI 0.95-1.26 p=0.2299) was not associated with 30 day mortality	Demographic factors, radiological factors and the ICH score.
Qureshi[20] 2011	ATACH I study	60	10 (16.7%)	Glucose trend over first 72 hours. All patients presented within 6 hours of symptom onset	90 day modified Rankin Score 4-6	Baseline glucose below median (115mg/dL) was not associated with outcome on both univariate (RR 1.44 95% CI 0.75-2.78, p not reported) or multivariable (RR 1.07 95% CI 0.78-1.48 p not reported) analyses<br>Declining glucose concentration over 72 hours was associated with poor outcome on univariate (RR 2.59 95% CI 1.27-5.30, p not reported) analysis but not statistically significant on multivariable analysis (RR 1.19 95% CI 0.92-1.54, p not reported)<br>Glucose reduction over 24 hours was not associated with outcome on both univariate (RR 1.56 95% CI 0.82-2.98 p not reported) and multivariable (RR 1.04 95% CI 0.53-2.05 p not reported) analyses.	GCS, ICH volume, IVH
Feng[21] 2012	USA	135	26 (19.3%)	Mean glucose within 72 hours. All patients presented within	90 day modified Rankin Score 3-6	Hyperglycemia (≥150mg/dL / 8.33 mMol/L) was not associated with poor outcome (OR 1.06 95% CI 0.4-2.66 p not reported)	Sex, mean arterial pressure, ICH score

				24 hours of symptom onset			
**Current study**	Finland	576	89 (15.5%)	Within 24 hours and between 24-72 hours. All patients presented within 24 hours of symptoms onset	6 month mortality	Persistent hyperglycemia (two measurement of >8mMol/L) was associated with 6 month mortality (OR 3.464 95% CI 1.868-6.424 p<0.001)	ICH volume, edema volume, NIHSS, GCS, IVH, warfarin use, age, anti-hypertensive use.

*Likely error in reporting 95% Confidence Interval in the published manuscript.
AVM indicates arteriovenous malformation; DNR, do not resuscitate; GCS, Glasgow Coma Scale; ICH, intracerebral hemorrhage; IVH, intraventricular hemorrhage; NIHSS, National Institutes of Health Stroke Scale; NR, not reported.

Supplemental References:

1. Franke CL, van Swieten JC, Algra A, van Gijn J. Prognostic factors in patients with intracerebral haematoma. *J Neurol Neurosurg Psychiatry*. 1992;55:653-657
2. Passero S, Ciacci G, Ulivelli M. The influence of diabetes and hyperglycemia on clinical course after intracerebral hemorrhage. *Neurology*. 2003;61:1351-1356
3. Fogelholm R, Murros K, Rissanen A, Avikainen S. Admission blood glucose and short term survival in primary intracerebral haemorrhage: A population based study. *J Neurol Neurosurg Psychiatry*. 2005;76:349-353
4. Kimura K, Iguchi Y, Inoue T, Shibazaki K, Matsumoto N, Kobayashi K, Yamashita S. Hyperglycemia independently increases the risk of early death in acute spontaneous intracerebral hemorrhage. *J Neurol Sci*. 2007;255:90-94
5. Tetri S, Juvela S, Saloheimo P, Pyhtinen J, Hillbom M. Hypertension and diabetes as predictors of early death after spontaneous intracerebral hemorrhage. *J Neurosurg*. 2009;110:411-417
6. Lee SH, Kim BJ, Bae HJ, Lee JS, Lee J, Park BJ, Yoon, BW. Effects of glucose level on early and long-term mortality after intracerebral haemorrhage: The acute brain bleeding analysis study. *Diabetologia*. 2010;53:429-434
7. Stead LG, Jain A, Bellolio MF, Odufuye A, Gilmore RM, Rabinstein A, Chandra R, Dhillon R, Manivannan V, Serrano LA, Yerragondu N, Palamari B, Jain M, Decker WW. Emergency department hyperglycemia as a

predictor of early mortality and worse functional outcome after intracerebral hemorrhage. *Neurocrit Care*. 2010;13:67-74

8. Samiullah S, Qasim R, Imran S, Mukhtair J. Frequency of stress hyperglycaemia and its' influence on the outcome of patients with spontaneous intracerebral haemorrhage. *J Pak Med Assoc*. 2010;60:660-663

9. Di Napoli M, Godoy DA, Campi V, del Valle M, Pinero G, Mirofsky M, Popa-Wagner A, Masotti L, Papa F, Rabinstein AA. C-reactive protein level measurement improves mortality prediction when added to the spontaneous intracerebral hemorrhage score. *Stroke*. 2011;42:1230-1236

10. Appelboom G, Piazza MA, Hwang BY, Carpenter A, Bruce SS, Mayer S, Connolly ES, Jr. Severity of intraventricular extension correlates with level of admission glucose after intracerebral hemorrhage. *Stroke*. 2011;42:1883-1888

11. Wang Y, Wang T, Zhang JH, Qin X. Effects of early serum glucose levels on prognosis of patients with acute intracerebral hemorrhage. *Acta Neurochir Suppl*. 2011;111:393-397

12. Bejot Y, Aboa-Eboule C, Hervieu M, Jacquin A, Osseby GV, Rouaud O, Giroud M. The deleterious effect of admission hyperglycemia on survival and functional outcome in patients with intracerebral hemorrhage. *Stroke*. 2012;43:243-245

13. Saxena A, Anderson CS, Wang X, Sato S, Arima H, Chan E, Munoz-Venturelli P, Delcourt C, Robinson T, Stapf C, Lavados PM, Wang J, Neal B, Chalmers J, Heeley E; INTERACT2 Investigators. Prognostic significance of

hyperglycemia in acute intracerebral hemorrhage: The INTERACT2 study. *Stroke*. 2016;47:682-688

14. Liu J, Wang D, Yuan R, Xiong Y, Liu M. Prognosis study of 908 patients with intracerebral hemorrhage in Chengdu, west of China. *Int J Neurosci*. 2016:1-18

15. Sun S, Pan Y, Zhao X, Liu L, Li H, He Y, Wang Y, Wang Y, Guo L. Prognostic value of admission blood glucose in diabetic and non-diabetic patients with intracerebral hemorrhage. *Sci Rep*. 2016;6:32342

16. Tapia-Perez JH, Gehring S, Zilke R, Schneider T. Effect of increased glucose levels on short-term outcome in hypertensive spontaneous intracerebral hemorrhage. *Clin Neurol Neurosurg*. 2014;118:37-43

17. Koga M, Yamagami H, Okuda S, Okada Y, Kimura K, Shiokawa Y, Nakagawara J, Furui E, Hasegawa Y, Kario K, Arihiro S, Sato S, Homma K, Matsuki T, Kinoshita N, Nagatsuka K, Minematsu K, Toyoda K; SAMURAI Study Investigators. Blood glucose levels during the initial 72 h and 3-month functional outcomes in acute intracerebral hemorrhage: The SAMURAI-ICH study. *J Neurol Sci*. 2015;350:75-78

18. Schwarz S, Hafner K, Aschoff A, Schwab S. Incidence and prognostic significance of fever following intracerebral hemorrhage. *Neurology*. 2000;54:354-361

19. Godoy DA, Pinero GR, Svampa S, Papa F, Di Napoli M. Hyperglycemia and short-term outcome in patients with spontaneous intracerebral hemorrhage. *Neurocrit Care*. 2008;9:217-229

20. Qureshi AI, Palesch YY, Martin R, Novitzke J, Cruz-Flores S, Ehtisham A, Ezzeddine MA, Goldstein JN, Kirmani JF, Hussein HM, Suri MF, Tariq N,

Liu Y; ATACH Investigators. Association of serum glucose concentrations during acute hospitalization with hematoma expansion, perihematomal edema, and three month outcome among patients with intracerebral hemorrhage. *Neurocrit Care*. 2011;15:428-435

21. Feng W, Tauhid S, Goel S, Sidorov EV, Selim M. Hyperglycemia and outcome in intracerebral hemorrhage: From bedside to bench-more study is needed. *Transl Stroke Res*. 2012;3:113-118
